# Urinary Diagnosis and Treatment

**Published:** 1901-05

**Authors:** 


### Book Reviews.

Urinary Diagnosis and Treatment. By J. W. Wainwright, M. D., Member of the American Medical Association, New York State Medical Association, New York County Medical Association, etc. Illustrated with numerous engravings and colored plates, 140 pages. Chicago: G P. Engelhard & Co. 1900. [Price, $1.00.]

The author's effort consists of about 118 pages of reading matter, several pages of test formulas and favorite prescriptions; also, about a dozen plates, these being in color.

Semen as a cause of opacity in freshly passed urine is not mentioned. It should have been, for at times this substance will be found to be a cause of marked turbidity, and this in the absence of any disease of the urinary organs. Wainwright fails to mention the quickest test for uric acid (not accurately quantitative, but amply so for ascertaining its presence) when in excess—namely, nitric acid placed below the urine with the development of a white band in the clear urine, about midway between the line of contact and the upper surface. He speaks several times regarding the amount of urea excreted as being important in the giving of a prognosis in certain conditions, but fails to mention the fact that as nitrogen is eliminated as urea, uric and hippuric acid, it is absolutely necessary that the amount of the two latter be known, for urea may be greatly diminished; whereas uric and hippuric acid may be present to an extent which will show that all nitrogen is being eliminated from the system.

Wainwright should not have failed to mention, even give prominence to, Purdy's test for albumin, for with this simple and quickly performed method, phosphates, mucin, and the like, are not coagulated, heat affecting the serum albumin only. We cannot accept the statement that uric acid crystals are always colored, for it is well known that one form, envelope shaped, are colorless (Tyson, Simon). Wainwright takes up diagnosis and treatment of diseases of the urinary organs, suggesting in the preface that his consideration of these is for the benefit of the busy practitioners, who have not the time to read several pages when sufficient information can be gained from a few words. If these assertions could be verified it would make that portion of the work very valuable, but they are not, and his opinion and advice is open to much criticism. For instance, in the care of acute nephritis no mention is made of any drug internally to diminish arterial tension; and the advice to use Basham's mixture in all conditions of chronic nephritis is an inexcusable error, for it is well known that in certain cases (plethora, for instance) iron is not only positively contraindicated, but it is deleterious. No mention is made of iodide of potash, mercury or glonoin, life-savers at times, and of inestimable value in many chronic conditions. To say if urine is passed in two or more receptacles, or obtained by a sterilised catheter, and the last contains bacteria, that cystitis is present, means simply that the writer is not acquainted with the anatomy or physiology of the urinary organs. In chronic posterior urethritis the last portion of urine will contain bacteria, even though it has been collected from the twelfth division.

Cystitis should be treated from the moment of onset by local medication and a cure obtained in a few hours instead of days or weeks, as would be the result if the author's advice was followed. It can scarcely be conceived why that most powerful agent, silver nitrate, receives no mention in the treatment of inflammations of this locality. Six pages of favorite prescriptions are given, many of which are valuable, but the author's attention must to be called to one for diabetes insipidus, page 124. This is probably a typographical error, but, nevertheless, it is a serious blunder to allow 80 grains of gallic acid to be advised as one dose, to be taken three or four times a day. Another supposed typographical error occurs on page 89, where Wainwright advises the use of a half inch oil immersion lens, no such an objective being made.

This work cannot be considered a pocket edition, as it is too large for one's pocket, but if the latter portion—diagnosis, treatment and favorite prescriptions—had been eliminated, it would have been a most valuable treatise for the physician who wants an epitomé on the urine. J. H. D.

